# Patient characteristics and health system encounters of decedents not marked deceased in the electronic health record

**DOI:** 10.1093/jamiaopen/ooae121

**Published:** 2024-10-29

**Authors:** Fernando Javier Sanz Vidorreta, Michael T Dudley, Anne M Walling, Chi-Hong Tseng, Michael Hogarth, Neil S Wenger

**Affiliations:** Biomedical Informatics Program at UCLA Clinical and Translational Science Institute, University of California, Los Angeles, CA 90095, United States; UCLA Office of Health Informatics and Analytics, University of California, Los Angeles, CA 90095, United States; UCLA Division of General Internal Medicine and Health Services Research, University of California, Los Angeles, CA 90024, United States; VA Greater Los Angeles Health System, Los Angeles, CA 90073, United States; UCLA Division of General Internal Medicine and Health Services Research, University of California, Los Angeles, CA 90024, United States; Division of Biomedical Informatics, Department of Medicine, University of California, San Diego, CA 92093, United States; UCLA Division of General Internal Medicine and Health Services Research, University of California, Los Angeles, CA 90024, United States

**Keywords:** vital status, fact-of-death, patient death, electronic health record

## Abstract

**Objectives:**

Health systems are increasingly accountable for patients and require accurate electronic health record (EHR) vital status. We recently demonstrated that 19% of seriously ill primary care patients in one system were not marked dead in the EHR and 80% of these decedents had an encounter or appointment outstanding after death. Herein we describe the mechanism of identifying decedents whose death is not captured at the level of the EHR, characterize these decedents, and describe medications refilled after death.

**Materials and Methods:**

Description of multistep process to identify deceased patients not marked dead in the EHR among a cohort of seriously ill primary care patients including public death file matching, utilization analysis, and chart abstraction. We compared decedents not marked dead in the EHR to known decedents and described pharmacy requests and refills.

**Results:**

Nearly 90% of encounters and appointments occurred because the health system EHR did not record the death although 11% of these encounters contained condolences or death notifications. Decedents not marked dead in the EHR were older and lived in more vulnerable areas than those marked dead. Of 146 refill requests after death, 88 medications were authorized.

**Discussion and Conclusion:**

Matching with a limited public death file is an inadequate solution to inaccurate vital status. Better workflows are needed to capture deaths about which clinicians and staff are aware, but will identify only a fraction of the decedents inaccurately listed as alive. Efforts are needed to connect EHRs with more specific sources of linkable decedent information.

## Background and significance

As health systems become increasingly accountable for the patients they treat and have greater responsibility for the comprehensive care they provide, it becomes increasingly important for health systems (and other health entities) to have access to up to date information about the vital status of their patients. Not knowing that patients have died has serious implications for health systems. If the health system is unaware that patients have died, population health management is less efficient because of unnecessary efforts to intervene on uncontrolled disease or missed preventive care for patients inaccurately thought to be still alive.^1^ Furthermore, those efforts may be insensitive to family and billing may be less efficient. Identifying high-priority patients for advance care planning and palliative care interventions also is more difficult if deaths are unrecognized. There also are implications for measurement when patients inappropriately remain in a measure’s denominator, for example in benchmarking colon cancer screening or vaccination rates.^2^

However, the vital status of seriously ill patients is too often incorrect in the electronic health record (EHR).^3^^,^^4^ This is because many patients die outside of healthcare institutions and there is no systematic way to capture that information. Because of the need to know that patients are dead, efforts have been made to integrate vital status into clinical data. These occur most often in the setting of clinical trials and often use national databases such as the National Death Index or Social Security Administration Death Master File (SSADMF),^5^^,^^6^ but these sources have a significant latency between death and the information appearing in the file and the SSADMF is missing a substantial number of deaths nationally.^4^^,^^7–11^ Furthermore, data elements such as Social Security Number have become less accessible in the EHR for matching because of security restrictions.^7^ We recently demonstrated that in one academic health system, 19% of seriously ill primary care patients were not marked dead in the EHR a mean of 19.8 months after death. Eighty percent of these decedents not recognized as dead by the EHR had an encounter or had an appointment outstanding after death. An average of 3.4 encounters and 11 elapsed appointment days accrued to patients erroneously thought to be alive.^12^

In this report, we describe the mechanism of identifying dead patients not marked dead in the EHR and investigate how decedents captured in the EHR compare to those not listed as dead. Furthermore, we characterize encounters after the date of death that are related to the patient’s death although the patient is not marked dead in the EHR. Lastly, for these patients not marked dead, we describe medications requested and refilled after the date of death.

## Methods

This study was conducted at one health system participating in a pragmatic trial of advance care planning conducted at the population level. Among patients considered to be alive in the health system EHR, we identified patients who were dead and then evaluated the characteristics of patients mistakenly categorized as alive and encounters with these decedents. The study was approved by the UCLA Institutional Review Board (18-001612).

### Patient population

The patient population was all patients aged 18 years or older who had a serious illness and who attended at least two primary care office visits during the prior 12 months in 41 clinics across a large academic health system. Serious illness was defined using administrative billing codes, encounter data, and clinical information available in the EHR. The definition of serious illness, validated by chart review, required an at-risk medical diagnosis (cancer, heart failure, chronic obstructive pulmonary disease, end-stage liver disease, end-stage renal disease, or amyotrophic lateral sclerosis) at a level of severity of illness such that advance care planning would be a priority, or one of these conditions linked with age 75 years or older.^13^ Patient demographic characteristics were obtained from the EHR. These included age, gender, race and ethnicity (White, Hispanic, Black, Asian and other), preferred language (English, Spanish and other), and insurance (commercial, Medicare, Medicaid, or other). We measured social vulnerability using the Social Vulnerability Index (SVI), which applies to the individual a level of vulnerability based on their home address that is derived from 15 US Census variables, such as income, educational level, employment, crowding, and vehicle access.^14^^,^^15^ SVI is an indicator of potential negative effects from external stress on health. An SVI score is attributed to each census tract ranging from 0 to 1, with 1 being the most vulnerable. Patient home addresses in our population were geocoded using ArcGIS Pro to obtain geographical coordinates. The coordinates were then binned into census tracts, which allowed SVI scores to be matched to each patient.

A baseline cohort of 6,607 patients was identified between the latter half of 2019 and when the clinics went live with the study intervention from January to June 2020. New seriously ill patients (N = 5,091) were accrued into the study cohort during the 24-month intervention period. Patients were followed for 2 years or until November 2022, whichever was earlier, for outcomes of advance directive completion and health care utilization. Death was recorded in the EHR by clinicians during routine clinical care according to usual health system protocol. Additional decedents were identified via returned mailed interventions and surveys, and these individuals were marked dead in the EHR.

### Analysis

We compared the seriously ill primary care study cohort alive according to the EHR against the California Department of Public Health Center for Health Statistics and Informatics Public Use Death File using a cut-off date of December 19, 2022. Matching was performed using an algorithm that calculates a score between 0 and 24 based on first and last name, middle initial, gender, and birthdate. The algorithm uses fuzzy logic for names and attributes a partial score for elements of birthdate. A score of 23 (at least an exact match on first and last names, birth date, and gender) was required for a match. The matched cohort was evaluated for clinical utilization after date of death (from the Public Use Death file) in order to identify outliers. We validated deaths by performing medical record abstraction on a random 10% of the matched decedent cases. For this validation, information about death was obtained from manual review of notes or documents in the EHR, from the linked electronic “Care Everywhere” record (information from other health systems linked electronically to the health system EHR) or by manual internet search for obituaries.

Among the patients not listed as dead in the EHR, we identified medical record encounters that occurred after the date of death until December 19, 2022. The content of each encounter was investigated by medical record abstraction of the 10% random sample of patients. This abstraction identified encounters that were related to the death (eg, notification by hospice, steps toward completion of a death certificate, condolence calls) or post-mortem disposition of paperwork or other materials. The proportion of death-related encounters within encounter type was used to estimate the frequency of non-death-related encounters in the full sample.^12^ For refill requests that were received after the date of death, we evaluated whether the medication was authorized and the type of medication.

Characteristics of decedents listed as dead in the EHR were compared to those not marked dead at the level of the EHR using chi square and *t*-tests. This comparison was performed in a multivariable fashion using logistic regression predicting unknown deaths. Computations used R and SAS statistical software; a *P*-value <.05 was considered statistically significant.

## Results

The 11,698 seriously ill primary care patients had a mean age of 75 years, 51% were male, 47% White, 13% Hispanic, 9% Asian, 8% Black, and 22% classified themselves to be of other race or ethnicity. Ninety percent spoke English, and mean SVI was 0.41 on the 0–1 scale. Of this seriously ill primary care patient cohort, 2,920 (25%) had death recorded in the EHR as of December 19, 2022 (a mean of 19.8 months after the date of death). Comparing the remaining patients marked as “alive” in the EHR against the California Department of Public Health Public Use Death File, we found that 677 patients matched a dead patient with a score of ≥23 (match of first and last name, gender and birth date). Review of utilization identified one outlier with 317 encounters after date of death compared to a mean of 3.9 encounters for the other 676 patients; this patient was confirmed alive on medical record abstraction. Thus, 676 of 3596 (19%) deaths among active primary care patients were not recognized in the health system EHR, as previously reported.^12^

Seventy-one of the 676 (10.5%) patients underwent medical record abstraction to evaluate vital status, and none was found to be alive. One patient was found to have an error in death date (by exactly 7 months), and a second patient had a death date within a few days of the CDPH Public Use Death File. For 42 (59%) patients, the exact date of death in the CDPH Public Use Death File was confirmed, for 13 patients (18%), the date of death was highly likely to be correct, and for 14 (20%) the date of death was probably correct (eg, a patient with a terminal condition on home hospice with no evidence of additional health care utilization). Method of confirmation of death and death date of the 10% chart abstracted sample (N = 71) is described in Table 1. This analysis shows that for 23 (32%) of the 71 decedents, information in the EHR (ranging from a clinician’s note to an outside hospital discharge summary) indicated the death.

**Table 1.** Medical record abstraction for confirmation of death and death date.

Method of confirmation	N
Date of death confirmed	42 (59%)
Death certificate in chart	3
Death note or discharge summary in chart	10
Chart note contains death date	10
Obituary	12
CareEverywhere death date*	7
Death confirmed, but not date of death	2 (3%)
Death date not exactly the same	1
Death date 7 months different	1
Date of death highly likely (eg, hospice, no additional care)	13 (18%)
Date of death probable (eg, severe illness, no additional care)	14 (20%)
**Total**	**71**

* CareEverywhere is an Epic link to outside health system records.

Comparing decedents not marked deceased in the EHR (N = 676) to patients with death recorded (N = 2920), patients not listed as deceased were older than the patients marked deceased in the EHR (mean age 79.9 v 78.1 years, *p* = .008) and had a different insurance composition with more Medicare insurance (*p* < .001) (Table 2).

**Table 2.** Comparison of characteristics of seriously ill primary care decedents marked deceased and not marked deceased in the EHR.

	Decedents marked deceased in the EHR (n = 2,920)	Decedents not marked deceased in the EHR (n = 676)	P value
Age, mean (SD)	78.1 (14.4)	79.9 (13.3)	*P* = .008
Gender, N (%)			*P* = .06
Male	1,569 (53.7%)	336 (49.7%)	
Female	1,351 (46.3%)	340 (50.3%)	
Race/ethnicity, N (%)			*P* = .6
White	1,705 (58.4%)	412 (60.9%)	
Hispanic	371 (12.7%)	79 (11.7%)	
Asian	250 (8.6%)	59 (8.7%)	
Black	245 (8.4%)	46 (6.8%)	
Other	349 (12.0%)	80 (11.8%)	
Language, N (%)			*P* = .10
English	2,620 (89.7%)	626 (92.6%)	
Spanish	156 (5.3%)	26 (3.8%)	
Other	144 (4.9%)	25 (3.7%)	
SVI, mean (SD)	0.40 (0.27)	0.41 (0.26)	*P* = .13
Insurance, N (%)			*P* < .001
Commercial	1,082 (37.1%)	225 (33.3%)	
Medicare	494 (16.9%)	171 (25.3%)	
Medicaid	540 (18.5%)	112 (16.6%)	
Other	371 (12.7%)	72 (10.7%)	
Missing	433 (14.8%)	96 (14.2%)	

EHR=electronic health record, SD=standard deviation.

SVI score ranges from 0 to 1, with 1 indicating higher vulnerability (N = 2263 for decedents marked dead in the EHR, and N = 535 for decedents not marked dead in the EHR).

Other race includes Native Hawaiian/Pacific Islander, American Indian/Alaskan Native, and multi-race/ethnicities.

In the multivariable model, compared to decedents marked dead in the EHR, decedents who were erroneously designated alive in the EHR were older (odds ratio [OR] 1.009, 95% confidence interval [95% CI] 1.002-1.016) and were more likely to live in an area with a higher SVI score (OR 1.56, 95% CI 1.05–2.32). Decedents not marked dead were also less likely to speak Spanish (OR 0.56, 95% CI 0.33–0.94), were less likely to be male (OR 0.83, 95% CI 0.70–0.99), and were more likely to have Medicare insurance (OR 1.58, 95% CI 1.26–1.99). There was no difference by race and ethnicity between groups (Table 3).

**Table 3.** Multivariable logistic regression of factors associated with decedent not recorded as dead in the electronic health record.

	Reference	Odds ratio (95% confidence interval)
**Age**	**Per year**	**1.009 (1.002–1.016)**
**Male**	**Female**	**0.83 (0.70–0.99)**
Hispanic	White	1.03 (0.74–1.43)
Asian	White	1.08 (0.79–1.48)
Black	White	0.71 (0.50–1.004)
Other race	White	1.00 (0.76–1.30)
**Spanish**	**English**	**0.56 (0.33–0.94)**
Other language	English	0.66 (0.41–1.05)
**SVI**	**0 to 1**	**1.56 (1.05–2.32)**
SVI missing	Not missing	1.10 (0.85–1.43)
**Medicare**	**Commercial**	**1.58 (1.26–1.99)**
Medicaid	Commercial	1.10 (0.83–1.45)
Other insurance	Commercial	0.89 (0.67–1.20)
Missing insurance	Commercial	1.04 (0.80–1.36)

**Bold** indicates *p* < .05.

Other race includes Native Hawaiian/Pacific Islander, American Indian/Alaskan Native, and multi-race/ethnicities.

Evaluation of each post-mortem encounter for the 71 patients in the chart abstraction sample revealed telephone encounters made by clinicians, staff, and care coordinators that were related to the death including obtaining information about the death, placing condolence calls, or arranging for paperwork to be completed or durable medical equipment to be retrieved. Calls unrelated to the death were placed as “wellness calls” to check on the patient, calls to schedule follow-up, or clinical calls to check on the progress of care. Five calls were placed by specialty clinics, advance care planning outreach, or for cancer registry follow-up. Overall, 52% of telephone calls after the date of death were related to the death. Two of the 21 (9.5%) patient portal messages were outreach inquiring about possible patient death. The other portal messages concerned clinical care or research. The two physician notes related to the patient’s death. Thus, overall, 30 (10.9%) of the 274 post-death encounters in the chart abstraction sample were related to the patient’s death, although for none of these patients was the death officially recorded in the EHR (Table 4).

**Table 4.** Health system and clinical outreach encounters after death (N = 71).

Encounter type	N	**N (%) related to death** *	N discounting those related to death
**Telephone outreach**	**50**	**26 (52%)**	**24**
Primary care or care coordinator	45	26	19
Cardiology	1	0	1
Dermatology	1	0	1
Advance care planning	2	0	2
Cancer registry	1	0	1
**Mailed letter outreach**	**106**	**0**	**106**
Flu shot	85	0	
Advance care planning	21	0	
**Patient portal outreach**	**21**	**2 (9.5%)**	**19**
Research	13	0	13
Primary care or care coordinator	4	2	2
Renal	2	0	2
Dermatology	2	0	2
**Note**	**2**	**2 (100%)**	**0**
**Orders placed**	**15**	**0**	**15**
COVID-19 vaccine	10	0	10
Retinal photos	3	0	3
Research	1	0	1
Clinical	1	0	1
**Refills**	**8**	**0**	**8**
**Appointments**	**72**	**0**	**72**
**Total**	**274**	**30 (10.9%)**	**244**

* Encounters related to death (row percentages shown) aimed at clarifying whether a patient was dead, completion of death certificates or other paperwork, and condolence calls.

Among the 676 dead patients not listed as dead, for 90 patients 130 medication refill requests were received after their date of death. The 130 refill requests contained requests for 146 different medications. Eighty-eight of the medications were authorized, and 57 were rejected; there was no response in 1 case. The medications included antihypertensive medications, thyroid replacement, hypoglycemics, anticoagulants, and many others (Supplementary Table 1). Reasons for rejecting a requested refill included the patient not having made a recent office visit, not having completed laboratory monitoring, or in some cases the clinician was aware that the patient was deceased (although the EHR did not reflect this).

## Discussion

Even among a cohort of seriously ill patients followed at least twice in the past year in primary care clinics, 19% were not marked dead in the EHR an average of 1½ years after death. This analysis shows that using a publicly available comprehensive state death file is an imperfect solution because matches on available demographic characteristics may incorrectly identify a patient as dead, which has substantial implications,^16^ and death dates are sometimes incorrect. Matching based on name, gender, and date of birth has limitations due to typographical errors in the data in one source or the other, and name matching is complicated by misspellings, use of abbreviations, name changes due to marriage or divorce, and use of nicknames. Name matching may be particularly problematic for vulnerable individuals.^17^ Manual medical record review also revealed that a proportion of decedents not marked dead in the EHR actually were known to be dead by a clinician or staff member within the health system, emphasizing the importance of efficient mechanisms to capture and validate this information so that it is apparent in the EHR. However, as shown in Table 4, the majority of health system interactions (outreach, held appointments, refill requests) occur in the setting of no EHR-level recording of the death, suggesting that this is not a problem isolated in one health system. This is not only wasteful but could harm families and reduce trust that health systems are aware of critical information.

Patients whose deaths were not recorded in the EHR, compared to those whose deaths were recorded, were older and more vulnerable. This is consistent with studies showing that lower SES and older adults in the United States were less likely to engage in eHealth activities^18^ and that vulnerable individuals had difficulty interacting with EHR patient portals.^19^ Yet, it is curious that women and decedents preferring Spanish language were associated with a greater likelihood that death information was captured and it is reassuring that race and ethnicity were not associated with missing information about death in the EHR. These demographic differences in death information capture provide little insight into the precise reasons that death was not recorded. Furthermore, it should be noted that decedents not marked dead in the EHR are a heterogeneous group, some known to be dead by health system personnel and most with no evidence that anyone in the health system was aware. Research is needed to identify each of the reasons that the death is not captured by the EHR, which may lead to potential solutions. Based on the abstracted sample of charts, these include streamlined mechanisms for health system staff to record death in the EHR, a mechanism to capture death from documents (eg, outside hospital discharge summaries) entered into the EHR, an electronic linkage with external health system death information, and notification from hospices and home health agencies.

Refill requests continued to arrive at the health system for these patients not marked deceased, presumably automatically generated by pharmacies that likely also were unaware that the patient was dead. We cannot know if authorized medications were retrieved by family members or others, but arguably^20^ they should not have been covered by insurance because pharmacy benefit manager companies likely have access to death data securely linkable at the individual patient level.

These data emphasize the importance of finding practical solutions to connecting EHR data with authoritative databases containing fact-of-death information. A major obstacle is that in many jurisdictions these more complete files (including social security number) are available “for purposes of law enforcement or preventing fraud”^21^ and are available to financial institutions, but not health care organizations. In these areas, legislative changes will be needed to permit use of statewide death files to update health data. A different solution could involve the fact-of-death service of the National Association for Public Health Statistics and Information Systems, which administers a real-time Electronic Verification of Vital Events.^22^ This system could serve this purpose as a national source of death information for health care systems, but has not been available to healthcare organizations and requires the availability of a SSN for matching.

Another approach would be to use the existing architecture used for linking patients for health data exchange across multiple healthcare organizations participating in a Health information exchange (HIE). This mechanism assigns a unique patient identifier that is not dependent on Social Security Number. If this informatics infrastructure could be used to link EHR records to the Electronic Death Registration System (EDRS) employing the data interoperability standards used in the national HIE networks, then patient vital status could be transmitted directly from EDRS in response to EHR queries. This could bypass state death record systems that appear to be unable to respond to such electronic inquiries. Using such a linkage, a national HIE infrastructure could create a seamless mechanism to match clinical records with death records. The idea of creating a unique national identification number is not new; the Health Insurance Portability and Accountability Act of 1996 instructs development of a unique identifier for patients.^23^ Concerns about confidentiality and other barriers have delayed development of a national patient identifier, suggesting that more collaboration with stakeholders is needed.^24^ This article illustrates one implication of not having an identifier to link clinical data systems with death information.

This analysis was performed in a single health system and may not be applicable to non-academic health systems or to health systems in venues with access to linkable death data. The analysis also may underestimate the magnitude of the problem because deaths were identified using a single state public use death file and patients may have died in other states. Furthermore, the patients were continuity primary care patients with serious illness that frequently interacted with providers and the healthcare system, which suggests that the proportion of unrecorded deaths is conservative. Additionally, it should be noted that the SVI is related to the area in which the patient lives and not the individual patient; thus, it is susceptible to the ecological fallacy and these findings should be interpreted with caution. Lastly, no harm was identified related to the health system interactions and refills; however, each point of contact is a potential source of harm and represents wasted health system resources.

## Conclusion

The EHR incorrectly labels nearly 1 in 5 decedents as alive with older and more vulnerable patients more likely to be mislabeled. A minority of this group could be captured by better EHR workflows, but publicly available data files are insufficient to correct death information for the majority. Legislative or IT solutions to improve EHR capture of patient death are needed.

## Supplementary Material

ooae121_Supplementary_Data

## Data Availability

Data are not available because analysis of the data for this clinical trial is ongoing.
