# Supplementary material for: Patient characteristics and health system encounters of decedents not marked deceased in the electronic health record
Source: JAMIA Open. 2024 Oct 29;7(4):ooae121. doi: 10.1093/jamiaopen/ooae121 (PMC11521374; doi:10.1093/jamiaopen/ooae121)
Supplement: ooae121_Supplementary_Data [file ooae121_supplementary_data.docx]

**SUPPLEMENTARY FILE**

**Table 1. Medications with Refill Requests after Death, Authorized and Rejected**

| **Medication** | **Refill Authorized** | **Refill Refused** |
| --- | --- | --- |
| abacavir | 1 | 0 |
| acyclovir | 0 | 1 |
| amiodarone | 1 | 2 |
| amlodipine | 3 | 2 |
| apixaban | 5 | 4 |
| atorvastatin | 5 | 5 |
| benazepril | 1 | 1 |
| budesonide-formoterol inhaler | 2 | 0 |
| bumetanide | 0 | 1 |
| bupropion | 1 | 0 |
| citalopram | 1 | 0 |
| cyclobenzaprine | 1 | 0 |
| digoxin | 1 | 6 |
| diltiazem | 1 | 0 |
| dulaglutide | 1 | 0 |
| escitalopram | 1 | 0 |
| ezetimibe-simvastatin | 1 | 0 |
| finasteride | 1 | 0 |
| fluoxetine | 1 | 1 |
| fluticasone-salmeterol | 2 | 0 |
| folate | 1 | 0 |
| furosemide | 7 | 7 |
| gentamycin ophthalmic solution | 0 | 1 |
| glipizide | 0 | 1 |
| hydrochlorothiazide | 1 | 0 |
| insulin | 1 | 1 |
| irbesartan | 0 | 1 |
| isosorbide | 1 | 0 |
| L-thyroxine | 8 | 3 |
| linaclotide | 1 | 0 |
| lisinopril | 0 | 1 |
| loratadine | 1 | 0 |
| lorazepam | 0 | 1 |
| losartan | 1 | 1 |
| megestrol | 1 | 0 |
| metformin | 4 | 0 |
| metoprolol | 5 | 3 |

| mirtazapine | 1 | 1 |
| --- | --- | --- |
| montelukast | 1 | 0 |
| multivitamin | 1 | 0 |
| naproxen | 1 | 0 |
| nifedipine | 1 | 0 |
| omeprazole | 2 | 3 |
| potassium chloride | 4 | 2 |
| pravastatin | 1 | 0 |
| prednisone | 0 | 2 |
| raltegravir | 1 | 0 |
| ropinirole | 1 | 0 |
| spironolactone | 2 | 0 |
| tamoxifen | 1 | 0 |
| tamsulosin | 0 | 1 |
| terazosin | 0 | 1 |
| thyroid | 0 | 1 |
| tiotropium inhaler | 1 | 0 |
| trazodone | 1 | 1 |
| trihexylphenidyl | 1 | 0 |
| timolol | 1 | 0 |
| venlafaxine | 2 | 0 |
| vitamin B12 | 1 | 0 |
| vitamin D | 0 | 1 |
| warfarin | 2 | 1 |
| **Total** | **88** | **57** |

No response to one refill request (for olanzapine).

The table describes refill authorizations and refusals for 146 refill requests for 90 patients.
